# Supplementary material for: Geographical distribution of freshwater fishes in Saudi Arabia
Source: PLoS One. 2024 Dec 13;19(12):e0311743. doi: 10.1371/journal.pone.0311743 (PMC11642914; doi:10.1371/journal.pone.0311743)
Supplement: S1 Table — (DOCX) [file pone.0311743.s001.docx]

Supplementary Table S1. The locations of water bodies

| Region | WBT | Species | Latitude | Longitude | location name | reference | Year | status |
| --- | --- | --- | --- | --- | --- | --- | --- | --- |
| EDS | Wadi | *Carasobarbus apoensis* | 18.28333474 | 42.56666934 | Khamis Mshait | Banister and Clarke | 1977 | indigenous |
|  |  | *Carasobarbus apoensis* | 22.93333469 | 40.90000217 | Wadi Turabah | Banister and Clarke | 1977 | indigenous |
|  |  | *Carasobarbus apoensis* | 19.88333472 | 41.95000228 | Wadi Adama | Banister and Clarke | 1977 | indigenous |
|  |  | *Carasobarbus apoensis* | 17.96666876 | 43.40000235 | Wadi al Mahallah | Alkahem and Behnke | 1983 | indigenous |
|  |  | *Carasobarbus apoensis* | 25.70000165 | 39.51666906 | Wadi Hadiyah | Alkahem and Behnke | 1983 | indigenous |
|  |  | *Carasobarbus apoensis* | 20.4833347 | 41.15000225 | Wadi Turabah | Krupp | 1983 | indigenous |
|  |  | *Carasobarbus apoensis* | 20.5000017 | 41.28333525 | Wadi Turabah | Krupp | 1983 | indigenous |
|  |  | *Carasobarbus apoensis* | 20.4833347 | 41.20000225 | Wadi Turabah | Krupp | 1983 | indigenous |
|  |  | *Carasobarbus apoensis* | 20.6500017 | 41.21666925 | Wadi shuqub | Krupp | 1983 | indigenous |
|  |  | *Carasobarbus apoensis* | 18.28333474 | 42.56666934 | Khamis Mshait | Krupp | 1983 | indigenous |
|  |  | *Carasobarbus apoensis* | 19.88333472 | 41.95000228 | Wadi Adama | Krupp | 1983 | indigenous |
|  |  | *Carasobarbus apoensis* | 20.4540907 | 41.19424626 | Wadi Turabah | Krupp | 1983 | indigenous |
|  |  | *Carasobarbus apoensis* | 20.5088537 | 41.26801925 | Wadi Turabah | Krupp | 1983 | indigenous |
|  |  | *Carasobarbus apoensis* | 19.92898372 | 41.69185228 | Wadi Al-janbeen | This study | - | indigenous |
|  |  | *Carasobarbus apoensis* | 20.13702472 | 41.71708927 | Wadi Tharad | This study | - | indigenous |
|  |  | *Carasobarbus apoensis* | 20.23672271 | 41.55131227 | Wadi Al-Aqiq | This study | - | indigenous |
|  |  | *Carasobarbus apoensis* | 20.7401377 | 41.01522924 | Wadi Buwah | This study | - | indigenous |
|  |  | *Carasobarbus apoensis* | 20.5406447 | 41.28562825 | Wadi Turabah | This study | - | indigenous |
|  |  | *Carasobarbus apoensis* | 20.6457597 | 41.22347925 | Wadi Shuqub | Krupp | 1983 | indigenous |
|  |  | *Carasobarbus apoensis* | 18.29153274 | 42.57209934 | Khamis Mushyat | Krupp | 1983 | indigenous |
|  |  | *Carasobarbus apoensis* | 19.89101572 | 41.95768328 | Wadi Adama | Krupp | 1983 | indigenous |
|  |  | *Carasobarbus apoensis* | 18.94947 | 42.53949 | Wadi Bishah | This study | - | indigenous |
|  |  | *Cyprinion acinaces* | 21.33333468 | 40.50000222 | near Ta'if | Banister and Clarke | 1977 | indigenous |
|  |  | *Cyprinion mhalense* | 20.4833347 | 41.15000225 | Wadi Turabah | Krupp | 1983 | indigenous |
|  |  | *Cyprinion mhalense* | 20.4833347 | 41.20000225 | Wadi Turabah | Krupp | 1983 | indigenous |
|  |  | *Cyprinion mhalense* | 20.5000017 | 41.28333525 | Wadi Turabah | Krupp | 1983 | indigenous |
|  |  | *Cyprinion mhalense* | 19.80000172 | 41.98333528 | Wadi Afrak | Krupp | 1983 | indigenous |
|  |  | *Cyprinion mhalense* | 19.68333473 | 42.06666929 | Wadi Adama | Krupp | 1983 | indigenous |
|  |  | *Cyprinion mhalense* | 20.6500017 | 41.21666925 | Wadi Shuqub | Krupp | 1983 | indigenous |
|  |  | *Cyprinion mhalense* | 20.7833347 | 41.20000224 | Wadi Buwah | Krupp | 1983 | indigenous |
|  |  | *Cyprinion mhalense* | 20.4333347 | 41.30000226 | Wadi Shumrukh | Krupp | 1983 | indigenous |
|  |  | *Cyprinion mhalense* | 18.23333474 | 42.58333534 | Wadi noaman | Krupp | 1983 | indigenous |
|  |  | *Cyprinion mhalense* | 21.33333468 | 40.35000222 | near Ta'if | Krupp | 1983 | indigenous |
|  |  | *Cyprinion mhalense* | 19.11666874 | 42.48333531 | Wadi Tarj | Hamidan and Shobrak | 2019 | indigenous |
|  |  | *Cyprinion mhalense* | 20.75000171 | 41.53333525 | Wadi Shumrukh | Hamidan and Shobrak | 2019 | indigenous |
|  |  | *Cyprinion mhalense* | 21.38333469 | 40.75000222 | Wadi Al-Arj | Hamidan and Shobrak | 2019 | indigenous |
|  |  | *Cyprinion mhalense* | 20.90000171 | 41.46666924 | Wadi Turabah | Hamidan and Shobrak | 2019 | indigenous |
|  |  | *Cyprinion mhalense* | 20.4560387 | 41.18965426 | Wadi Turabah | Krupp | 1983 | indigenous |
|  |  | *Cyprinion mhalense* | 20.5406447 | 41.28562825 | Wadi Turabah | This study |  | indigenous |
|  |  | *Cyprinion mhalense* | 19.92898372 | 41.69185228 | Wadi Al-janbeen | This study |  | indigenous |
|  |  | *Cyprinion mhalense* | 20.13702472 | 41.71708927 | Wadi Tharad | This study |  | indigenous |
|  |  | *Cyprinion mhalense* | 20.23672271 | 41.55131227 | Wadi Al-Aqiq | This study |  | indigenous |
|  |  | *Cyprinion mhalense* | 20.6156337 | 41.20464625 | Wadi Shuqub | Krupp | 1983 | indigenous |
|  |  | *Cyprinion mhalense* | 20.7593537 | 41.18226625 | Wadi Buwah | Krupp | 1983 | indigenous |
|  |  | *Cyprinion mhalense* | 20.7398567 | 41.01403824 | Wadi Buwah | This study | - | indigenous |
|  |  | *Cyprinion mhalense* | 20.4386087 | 41.29881126 | Wadi Shumrukh | Krupp | 1983 | indigenous |
|  |  | *Cyprinion mhalense* | 18.30372774 | 42.58612734 | Wadi Al-Mahallah | Alkahem and Behnke | 1983 | indigenous |
|  |  | *Cyprinion mhalense* | 18.30372774 | 42.58612734 | Wadi Al-Mahallah | Krupp | 1983 | indigenous |
|  |  | *Cyprinion mhalense* | 21.33885768 | 40.57131022 | Taif | Krupp | 1983 | indigenous |
|  |  | *Cyprinion mhalense* | 18.94947 | 42.53949 | Wadi Bishah | This study | - | indigenous |
|  |  | *Garra buettikeri* | 20.4833347 | 41.20000225 | Wadi Turabah | Krupp | 1983 | indigenous |
|  |  | *Garra buettikeri* | 20.5000017 | 41.28333525 | Wadi Turabah | Krupp | 1983 | indigenous |
|  |  | *Garra buettikeri* | 19.43333473 | 42.0500023 | Wadi Adama | Krupp | 1983 | indigenous |
|  |  | *Garra buettikeri* | 19.68333473 | 42.06666929 | Wadi Adama | Krupp | 1983 | indigenous |
|  |  | *Garra buettikeri* | 20.3833347 | 41.31666926 | Wadi Noval | Krupp | 1983 | indigenous |
|  |  | *Garra buettikeri* | 20.4500017 | 41.31666926 | Wadi Shumrukh | Krupp | 1983 | indigenous |
|  |  | *Garra buettikeri* | 18.23333474 | 42.58333534 | Wadi Noaman | Krupp | 1983 | indigenous |
|  |  | *Garra buettikeri* | 20.75000171 | 41.53333525 | Wadi Shumrukh | Hamidan and Shobrak | 2019 | indigenous |
|  |  | *Garra buettikeri* | 21.38333469 | 40.75000222 | Wadi Al-Arj | Hamidan and Shobrak | 2019 | indigenous |
|  |  | *Garra buettikeri* | 20.90000171 | 41.46666924 | Wadi Turabah | Hamidan and Shobrak | 2019 | indigenous |
|  |  | *Garra buettikeri* | 20.5030087 | 41.27629525 | Wadi Turabah | Krupp | 1983 | indigenous |
|  |  | *Garra buettikeri* | 19.68333473 | 42.06666929 | Wadi Adama | Krupp | 1983 | indigenous |
|  |  | *Garra buettikeri* | 20.5221317 | 41.27150825 | Wadi Turabah | This study | - | indigenous |
|  |  | *Garra buettikeri* | 20.13702472 | 41.71708927 | Wadi Tharad | This study | - | indigenous |
|  |  | *Garra buettikeri* | 20.7379287 | 41.02710524 | Wadi Buwah | This study | - | indigenous |
|  |  | *Garra buettikeri* | 20.3833347 | 41.31666926 | Wadi Noval | Krupp | 1983 | indigenous |
|  |  | *Garra buettikeri* | 20.4685747 | 41.30409426 | Wadi Shumrukh | Krupp | 1983 | indigenous |
|  |  | *Garra buettikeri* | 20.23672271 | 41.55131227 | Wadi Al-Aqiq | This study | - | indigenous |
|  |  | *Garra buettikeri* | 19.92898372 | 41.69185228 | Wadi Al-janbeen | This study | - | indigenous |
|  |  | *Oreochromis niloticus* | 18.95319 | 42.53646 | Wadi Bishah | This study | - | introduced |
|  | reservoir | *Carasobarbus apoensis* | 19.90239972 | 41.71063628 | Al-janbeen Dam | This study | - | indigenous |
|  |  | *Carasobarbus apoensis* | 20.15858472 | 41.72127827 | Tharad Dam | This study | - | indigenous |
|  |  | *Carasobarbus apoensis* | 20.23705871 | 41.57139827 | Al-Aqiq Dam | This study | - | indigenous |
|  |  | *Carasobarbus apoensis* | 20.12595371 | 41.35888327 | AL-Sdar,Wadi and Dam | This study | - | indigenous |
|  |  | *Carasobarbus apoensis* | 20.2227347 | 41.27415926 | Medhaas Dam | This study | - | indigenous |
|  |  | *Cyprinion mhalense* | 20.2227347 | 41.27415926 | Medhaas Dam | This study | - | indigenous |
|  |  | *Cyprinion mhalense* | 19.90239972 | 41.71063628 | Al-janbeen Dam | This study | - | indigenous |
|  |  | *Cyprinion mhalense* | 20.15858472 | 41.72127827 | Tharad Dam | This study | - | indigenous |
|  |  | *Cyprinion mhalense* | 20.23705871 | 41.57139827 | Al-Aqiq Dam | This study | - | indigenous |
|  |  | *Cyprinion mhalense* | 20.12595371 | 41.35888327 | AL-Sdar,Wadi and Dam | This study | - | indigenous |
|  |  | *Cyprinion mhalense* | 18.21497 | 42.48526 | Abha Dam | This study | - | indigenous |
|  |  | *Garra buettikeri* | 20.2222507 | 41.27556826 | Medhaas Dam | This study | - | indigenous |
|  |  | *Garra buettikeri* | 19.91104772 | 41.71129528 | Al-janbeen Dam | This study | - | indigenous |
|  |  | *Garra buettikeri* | 20.16458472 | 41.71649127 | Tharad Dam | This study | - | indigenous |
|  |  | *Garra buettikeri* | 20.24124771 | 41.56322127 | Al-Aqiq Dam | This study | - | indigenous |
|  |  | *Garra buettikeri* | 20.12595371 | 41.35888327 | AL-Sdar,Wadi and Dam | This study | - | indigenous |
|  |  | *Garra buettikeri* | 18.21666874 | 42.48333534 | Abha Dam | Krupp | 1983 | indigenous |
|  |  | *Orechromis niloticus* | 20.15758472 | 41.72222427 | Tharad Dam | This study | - | introduced |
|  |  | *Orechromis niloticus* | 18.21666874 | 42.48333534 | Abha Dam | This study | - | introduced |
|  |  | *Orechromis niloticus* | 19.69925 | 42.48413 | King Fahad Dam | This study | - | introduced |
|  |  | *Orechromis niloticus* | 19.74744 | 41.98857 | Alfouha Dam | This study | - | introduced |
|  |  | *Orechromis niloticus* | 17.90333 | 42.38532 | Marabah Dam | This study | - | introduced |
|  |  | *Carassius auratus* | 19.90350672 | 41.71161328 | Al-janbeen Dam | This study | - | introduced |
|  |  | *Carassius auratus* | 18.21071174 | 42.48751134 | Abha Dam | This study | - | introduced |
|  |  | *Carassius auratus* | 20.2138857 | 41.27591426 | near Medhaas | This study | - | introduced |
|  |  |  |  |  |  |  |  |  |
| WDS  WDS | Wadi  Wadi | *Carasobarbus apoensis* | 22.81666865 | 39.36666916 | Wadi Haqqaq | Borkenhagen and Krupp | 2013 | indigenous |
|  |  | *Carasobarbus apoensis* | 25.56666863 | 38.68333505 | Wadi Hadiyah | Borkenhagen and Krupp | 2013 | indigenous |
|  |  | *Arabibarbus arabicus* | 17.333333 | 42.133333 | Wadi Juva | Krupp | 1983 | indigenous |
|  |  | *Arabibarbus arabicus* | 17.33333475 | 43.03333537 | Wadi Damad | Hamidan and Shobrak | 2019 | indigenous |
|  |  | *Arabibarbus arabicus* | 17.58604474 | 42.61831036 | Wadi Baish | Hakami | 2013 | indigenous |
|  |  | *Cyprinion acinaces* | 25.70000164 | 39.20000205 | Khaibar | Krupp | 1983 | indigenous |
|  |  | *Cyprinion acinaces* | 25.60000164 | 39.26666906 | Wadi Sulaym | Krupp | 1983 | indigenous |
|  |  | *Cyprinion acinaces* | 24.00000164 | 39.00000211 | Wadi Hadiyah | Krupp | 1983 | indigenous |
|  |  | *Cyprinion acinaces* | 25.70000164 | 39.20000205 | close to Kaibar | Krupp | 1983 | indigenous |
|  |  | *Cyprinion acinaces* | 25.60000164 | 39.26666906 | Wadi Sulaym ,Kaibar | Krupp | 1983 | indigenous |
|  |  | *Garra buettikeri* | 18.77000172 | 41.98000232 | Wadi Al-Bagarah | Hamidan and Shobrak | 2019 | indigenous |
|  |  | *Acanthobrama hadiyahensis* | 25.550002 | 38.733335 | Wadi Hadiyah | Coad et al | 1983 | indigenous |
|  |  | *Acanthobrama hadiyahensis* | 25.547724 | 38.813584 | Wadi Hadiyah | Al-Kahem | 2004 | indigenous |
|  |  | *Garra sahilia* | 20.51666869 | 40.66666925 | Wadi Minsah | Krupp | 1983 | indigenous |
|  |  | *Garra sahilia* | 17.53333474 | 42.41666936 | Wadi North Jizan | Krupp | 1983 | indigenous |
|  |  | *Garra sahilia* | 19.70000171 | 41.40000228 | Al-Ahsabah Dam | Krupp | 1983 | indigenous |
|  |  | *Garra sahilia* | 18.43333472 | 41.88333533 | Wadi Gaanah | Krupp | 1983 | indigenous |
|  |  | *Garra sahilia* | 19.15000172 | 41.8200023 | Wadi Kudais | Hamidan and Shobrak | 2019 | indigenous |
|  |  | *Garra sahilia* | 18.72000172 | 41.99000232 | Wadi Al-Gassah | Hamidan and Shobrak | 2019 | indigenous |
|  |  | *Garra sahilia* | 18.77000172 | 41.98000232 | Wadi Al-Bagarah | Hamidan and Shobrak | 2019 | indigenous |
|  |  | *Garra sahilia* | 17.46000175 | 42.88000236 | Wadi Haroub | Hamidan and Shobrak | 2019 | indigenous |
|  |  | *Garra sahilia* | 20.51853169 | 40.66943525 | Wadi Minsah | Krupp | 1983 | indigenous |
|  |  | *Garra sahilia* | 17.57297874 | 42.42488336 | N of Jizan | Krupp | 1983 | indigenous |
|  |  | *Garra sahilia* | 20.10819869 | 40.86772826 | Wadi ileeb | Krupp | 1983 | indigenous |
|  |  | *Garra tibanica* | 17.53333474 | 42.41666936 | Wadi North Jizan | Banister and Clarke | 1977 | indigenous |
|  |  | *Garra tibanica* | 25.70000164 | 39.20000205 | Khaibar | Krupp | 1983 | indigenous |
|  |  | *Garra tibanica* | 18.08333473 | 42.35000234 | Wadi Hesu'a | Krupp | 1983 | indigenous |
|  |  | *Garra tibanica* | 17.28333476 | 43.10000237 | Wadi Damad | Krupp | 1983 | indigenous |
|  |  | *Garra tibanica* | 17.33333475 | 43.03333537 | Wadi Damad | Hamidan and Shobrak | 2019 | indigenous |
|  |  | *Garra tibanica* | 23.10000166 | 39.70000215 | Wadi Khadrah | Hamidan and Shobrak | 2019 | indigenous |
|  |  | *Garra tibanica* | 21.79138367 | 40.0074342 | Wadi Fatima | Krupp | 1983 | indigenous |
|  |  | *Garra tibanica* | 17.20427975 | 43.00261337 | Wadi Damad | Krupp | 1983 | indigenous |
|  |  | *Garra tibanica* | 17.63799874 | 42.39033335 | Wadi Juva | Krupp | 1983 | indigenous |
|  |  | *Garra tibanica* | 18.08490073 | 42.35028434 | Wadi Hesu'a | Krupp | 1983 | indigenous |
|  |  | *Garra tibanica* | 25.67773264 | 39.21192205 | Khaibar | Krupp | 1983 | indigenous |
|  |  | *Aphaniops dispar* | 16.76000176 | 43.12000239 | Wadi Khulab | Hamidan and Shobrak | 2019 | secondary |
|  |  | *Aphaniops dispar* | 18.77000172 | 41.98000232 | Wadi Al-Bagarah | Hamidan and Shobrak | 2019 | secondary |
|  |  | *Aphaniops dispar* | 19.15000172 | 41.8200023 | Wadi Kudais | Hamidan and Shobrak | 2019 | secondary |
|  |  | *Aphaniops dispar* | 23.10000166 | 39.70000215 | Wadi Khadrah | Hamidan and Shobrak | 2019 | secondary |
|  |  | *Aphaniops dispar* | 21.69836266 | 39.8871412 | Wadi Fatima | Krupp | 1983 | secondary |
|  |  | *Aphaniops dispar* | 17.78271073 | 42.14012635 | Wadi Reem | Krupp | 1983 | secondary |
|  |  | *Orechromis niloticus* | 22.74369564 | 39.19253016 | Wadi Rabig | This study | - | introduced |
|  |  | *Aphaniops dispar* | 22.74369564 | 39.19253016 | Wadi Rabig | This study | - | secondary |
|  |  | *Oreochromis mossambicus* | 25.52901345 | 38.80580831 | Wadi Hadiyah | This study | - | introduced |
|  | reservoir | *Arabibarbus arabicus* | 17.66740374 | 42.65953436 | Baish Dam | Hakami | 2013 | indigenous |
|  |  | *Acanthobrama hadiyahensis* | 25.484514 | 39.362822 | Al-Bint Dam | Hamidan and Shobrak | 2019 | indigenous |
|  |  | *Acanthobrama hadiyahensis* | 25.203333 | 39.363194 | Khaibar,Qusaiba’a Dam | Hamidan and Aloufi | 2014 | indigenous |
|  | spring | *Cyprinion acinaces* | 25.78333465 | 39.43333505 | Ain Al-Hammah | Hamidan and Shobrak | 2019 | indigenous |
|  |  | *Garra sahilia* | 20.46000168 | 40.46000225 | Alein Al-Harrah | Hamidan and Shobrak | 2019 | indigenous |
|  |  | *Garra tibanica* | 25.78333465 | 39.43333505 | Ain Al-Hammah | Hamidan and Shobrak | 2019 | indigenous |
|  |  | *Aphaniops dispar* | 25.72000164 | 39.26000205 | Ain Al-Buhairah | Hamidan and Shobrak | 2019 | secondary |
| EP | Irrigation channels | *Aphaniops stoliczkanus* | 26.55000193 | 50.00000219 | al-Qatif | Krupp | 1983 | secondary |
|  |  | *Aphaniops stoliczkanus* | 25.40000192 | 49.46666921 | al-Hufuf | Krupp | 1983 | secondary |
|  |  | *Aphaniops stoliczkanus* | 25.43275192 | 49.61403022 | al-Hasa oasis | Krupp | 1983 | secondary |
|  |  | *Orechromis niloticus* | 25.36262292 | 49.68327422 | al-Hasa oasis | This study | - | introduced |
|  |  | *Gambusia holbrooki* | 25.40517992 | 49.73533722 | al-Hasa canals | This study | - | introduced |
|  |  | *Poecilia latipinna* | 25.39322992 | 49.71357722 | al-Hasa oasis | This study | - | introduced |
|  |  | *Ctenopharyngodon idella* | 25.46964592 | 49.62541921 | al-Hasa canals | Almutairi | - | introduced |
|  |  | *Planiliza abu* | 25.46614092 | 49.59491921 | al-Hasa oasis | Al-Kahem | 2004 | Complementary |
|  |  | *Cyprinus carpio* | 25.409671 | 49.62569 | al-Hasa oasis | Almutairi | - | introduced |
|  | LAKE | *Orechromis niloticus* | 25.51905792 | 49.80521322 | al-Asfer Lake | This study | - | introduced |
|  |  | *Gambusia holbrooki* | 25.53867792 | 49.81192822 | al-Asfer Lake | This study | - | introduced |
|  | spring | *Aphaniops stoliczkanus* | 25.42200192 | 49.58716922 | Khodod spring | Krupp | 1983 | secondary |
|  |  | *Aphaniops stoliczkanus* | 25.43275192 | 49.61403022 | al-Hasa oasis | Krupp | 1983 | secondary |
| MR | LAKE | *Orechromis niloticus* | 25.90070281 | 45.32662814 | al-Majmaah | This study | - | introduced |
|  |  | *Carassius auratus* | 24.61933485 | 46.7085302 | Salam Lake | This study | - | introduced |
|  |  | *Cyprinus carpio* | 24.618956 | 46.707847 | Salam Lake | This study | - | introduced |
|  | reservoir | *Orechromis niloticus* | 24.57020485 | 46.6731312 | Nemar Dam | This study | - | introduced |
|  |  | *Poecilia latipinna* | 24.57481085 | 46.6856822 | Nemar Dam | This study | - | introduced |
|  |  | *claris gariepinus* | 24.57189085 | 46.6821412 | Nemar Dam | This study | - | introduced |
|  |  | *Carassius auratus* | 24.57036285 | 46.6744192 | Nemar Dam | This study | - | introduced |
|  | Wadi | *Aphaniops stoliczkanus* | 24.35000186 | 47.18333521 | al-Kharj | Krupp | 1983 | secondary |
|  |  | *Aphaniops stoliczkanus* | 24.63352685 | 46.65759019 | Wadi Hanefah | Al-Kahem | 2004 | secondary |
|  |  | *Orechromis niloticus* | 24.13208687 | 47.40081422 | al-Kharj | Al-Kahem | 2004 | introduced |
|  |  | *Orechromis niloticus* | 24.53419085 | 46.7591412 | al-Kharj | Al-Kahem | 2004 | introduced |
|  |  | *Orechromis niloticus* | 24.41897385 | 46.8143162 | al-Hair,Riyadh | This study |  | introduced |
|  |  | *Orechromis aurius* | 24.38380785 | 46.8297652 | al-Hair,Riyadh | Al-Kahem | 2004 | introduced |
|  |  | *Poecilia reticulata* | 24.56887785 | 46.6728232 | Wadi Hanefah | Al-Kahem | 2004 | introduced |
|  |  | *xiphiphorus maculatus* | 24.55194185 | 46.7385682 | Wadi Hanefah | Al-Kahem | 2004 | introduced |
|  |  | *claris gariepinus* | 24.38534885 | 46.8452402 | Wadi Hanefah | This study | - | introduced |
|  |  | *Orechromis niloticus* | 26.18545278 | 44.00864211 | Wadi Rumah | This study | - | introduced |
| MRN | Wadi | *claris gariepinus* | 26.18545278 | 44.00864211 | Wadi Rumah | This study | - | introduced |
| NORTH | LAKE | *Gambusia holbrooki* | 29.81423066 | 39.91068591 | Duma Jandal Lake | This study | - | introduced |
