# Supplementary material for: Geographical distribution of freshwater fishes in Saudi Arabia
Source: PLoS One. 2024 Dec 13;19(12):e0311743. doi: 10.1371/journal.pone.0311743 (PMC11642914; doi:10.1371/journal.pone.0311743)
Supplement: Attachment — Submitted filename: Response to Reviewers 5-9-2024.docx [file pone.0311743.s003.docx]

Dear Dr. Alharthi,

**Editor:** Thank you for submitting your manuscript to PLOS ONE. After careful consideration, we feel that it has merit but does not fully meet PLOS ONE’s publication criteria as it currently stands. Therefore, we invite you to submit a revised version of the manuscript that addresses the points raised during the review process.

**Author:** Dear Editor and Reviewers, Thank you for your valuable comments. We have revised the manuscript according to the highlighted shortcomings.

We look forward to receiving your revised manuscript.

Kind regards,

Benigno Elvira, Ph.D.

Academic Editor

PLOS ONE

**Journal Requirements**

When submitting your revision, we need you to address these additional requirements.

1. Please ensure that your manuscript meets PLOS ONE's style requirements, including those for file naming. The PLOS ONE style templates can be found at

https://journals.plos.org/plosone/s/file?id=wjVg/PLOSOne_formatting_sample_main_body.pdf and

https://journals.plos.org/plosone/s/file?id=ba62/PLOSOne_formatting_sample_title_authors_affiliations.pdf

**Author:** Completely updated according to the formats.

2. Thank you for stating the following financial disclosure:

"National Centre for Wildlife for providing PhD scholarship to Ibrahim G. Alharthi including financial support for the study."

Please state what role the funders took in the study. If the funders had no role, please state: ""The funders had no role in study design, data collection and analysis, decision to publish, or preparation of the manuscript.""

If this statement is not correct you must amend it as needed.

Please include this amended Role of Funder statement in your cover letter; we will change the online submission form on your behalf.

**Author:** Changes done.

3. Thank you for stating the following in the Acknowledgments Section of your manuscript:

"We are grateful for the support and assistance of numerous individuals and organizations in the completion of this study. I would like to thank the National Centre for Wildlife of (the Kingdom of Saudi Arabia for permission to conduct this research and for providing the necessary resources and facilities, special thanks to Mr. Muhammad Al-Nashiri from GIS department for his invaluable contribution in enriching and improving the quality of maps. Thanks are extended to the University of Hull International Fisheries Institute staff for supporting my research."

**Author:** Ok.

We note that you have provided funding information that is currently declared in your Funding Statement. However, funding information should not appear in the Acknowledgments section or other areas of your manuscript. We will only publish funding information present in the Funding Statement section of the online submission form.

Please remove any funding-related text from the manuscript and let us know how you would like to update your Funding Statement. Currently, your Funding Statement reads as follows:

"National Centre for Wildlife for providing PhD scholarship to Ibrahim G. Alharthi including financial support for the study."

Please include your amended statements within your cover letter; we will change the online submission form on your behalf.

**Author:** Changes done.

4. We note that Figures 1, 2, 5 and Supporting Information in your submission contain map/satellite images which may be copyrighted. All PLOS content is published under the Creative Commons Attribution License (CC BY 4.0), which means that the manuscript, images, and Supporting Information files will be freely available online, and any third party is permitted to access, download, copy, distribute, and use these materials in any way, even commercially, with proper attribution. For these reasons, we cannot publish previously copyright maps or satellite images created using proprietary data, such as Google software (Google Maps, Street View, and Earth). For more information, see our copyright guidelines: http://journals.plos.org/plosone/s/licenses-and-copyright.

**Author:** Maps have been changed for this research paper and has no copyright anymore

We require you to either (1) present written permission from the copyright holder to publish these figures specifically under the CC BY 4.0 license, or (2) remove the figures from your submission:

**Author:** We did not include any copyright material in our manuscript.

a. You may seek permission from the original copyright holder of Figures 1, 2, 5 and Supporting Information to publish the content specifically under the CC BY 4.0 license.

**Author:** We did not include any copyright material in our manuscript.

We recommend that you contact the original copyright holder with the Content Permission Form (http://journals.plos.org/plosone/s/file?id=7c09/content-permission-form.pdf) and the following text:

“I request permission for the open-access journal PLOS ONE to publish XXX under the Creative Commons Attribution License (CCAL) CC BY 4.0 (http://creativecommons.org/licenses/by/4.0/). Please be aware that this license allows unrestricted use and distribution, even commercially, by third parties. Please reply and provide explicit written permission to publish XXX under a CC BY license and complete the attached form.”

**Author:** Maps have been changed for this research paper and has no copyright anymore.

Please upload the completed Content Permission Form or other proof of granted permissions as an ""Other"" file with your submission.

**Author:** Maps have been changed for this research paper and has no copyright anymore. In the figure caption of the copyrighted figure, please include the following text: “Reprinted from [ref] under a CC BY license, with permission from [name of publisher], original copyright [original copyright year].”

**Author:** Maps have been changed for this research paper and has no copyright anymore

b. If you are unable to obtain permission from the original copyright holder to publish these figures under the CC BY 4.0 license or if the copyright holder’s requirements are incompatible with the CC BY 4.0 license, please either i) remove the figure or ii) supply a replacement figure that complies with the CC BY 4.0 license. Please check copyright information on all replacement figures and update the figure caption with source information. If applicable, please specify in the figure caption text when a figure is similar but not identical to the original image and is therefore for illustrative purposes only.

The following resources for replacing copyrighted map figures may be helpful:

**Author:** Maps have been changed for this research paper and has no copyright anymore.

USGS National Map Viewer (public domain): http://viewer.nationalmap.gov/viewer/

The Gateway to Astronaut Photography of Earth (public domain): http://eol.jsc.nasa.gov/sseop/clickmap/

Maps at the CIA (public domain): https://www.cia.gov/library/publications/the-world-factbook/index.html and https://www.cia.gov/library/publications/cia-maps-publications/index.html

NASA Earth Observatory (public domain): http://earthobservatory.nasa.gov/

Landsat: http://landsat.visibleearth.nasa.gov/

USGS EROS (Earth Resources Observatory and Science (EROS) Center) (public domain): http://eros.usgs.gov/#

Natural Earth (public domain): http://www.naturalearthdata.com/

5. Please include captions for your Supporting Information files at the end of your manuscript, and update any in-text citations to match accordingly. Please see our Supporting Information guidelines for more information: http://journals.plos.org/plosone/s/supporting-information.

**Review Comments to the Author**

**Reviewer #1**

This is a nice manuscript and I see no major obstacles to publish this. Indeed, old data had been compiled for that book - https://www.researchgate.net/publication/341322582_Freshwater_Fishes_of_the_Arabian_Peninsula ignored in the manuscript, and replaced by a checklist, which just copies the book/steals the information. Maybe not nice.

**Authors:** Dear Reviewer, thank you for your comments. We did not compile any book data of the previous record. Nor we followed this mentioned book. We gather the information from the sampling and provided in the checklist. Furthermore, this publication part of my PhD which completed 2019 which is before the book. Besides, all distribution coordinates available at many papers which I believe the book also used same data as me.

I have two points - I do not believe that Carassius carassius was introduced and your records must be based on misidentification - please check again and write a short sentece why - and show a picture of such fish. The identification of C. gibelio is likely also wrong - and all are likely to be C. auratus. Please explain some things.

**Authors:** Dear Reviewer, I believe Carassius carassius should be removed as you stated. My first catch appeared to be a Crucian carp, based on my initial assessment. However, I reviewed this and it was a misidentification and the species is actually a goldfish (C. auratus). As shown in the picture below, these two species share many characteristics, so I worked on updated maps and text based on this.

Furthermore, I like to see some text how you identified the Oreochromis - this is not trivial and maybe here also you show some pictures.

**Authors:** It is not easy to identify Oreochromis as you mentioned, I used a reference at that time to flow identification keys such as dorsal spine and soft rays etc. which also not easy due to their number intersects among species and hybridization, I will try to be added this into text. Here some picture

 Last but not least,

Please add a table to the suppl. materials in which you make clear, which species you have found at which place - this is only for your own work. Basically an excel with a line for each record: species/coordinates/date..... is the way. By this you make your new records available

**Authors:**  Dear Reviewer, thank you for this insightful suggestion, the supplementary file has been updated with your suggestion.

**Reviewer #2**

The authors document and analyse the distribution of inland water fishes in Saudi Arabia, based on literature records and field surveys, adding new and unpublished data. The paper will be of great importance for inland water fish conservation efforts in the Kingdom of Saudi Arabia and deserves to be published. Here below, I am offering extensive comments, which I trust will help the authors to revise the paper.

**Authors:** Dear Reviewer, it is very much valuable for your comments and appreciation for the publication of this study. We have revised the manuscript against each of your comment and responded in the response letter.

My major concerns with the current draft of the paper are as follows: The title reads “Biogeography of freshwater fishes in Saudi Arabia”. Biogeography is defined as “the study of the distribution of organisms across geographic space, and the processes that shape these distributions”. This paper reports the geographic distribution but does not analyze the processes that shape these distributions. A key factor in shaping the distribution of native inland water fishes is the geological history of the drainage basins, which is not included in this paper. For this reason, I would not consider this a biogeographic study. Biogeographic studies only make sense for native species.

**Authors:** The title has been re-written with the concept of geographical distribution of freshwater fishes. The biogeography has been omitted.

The paper would benefit from a clearer distinction between native primary and secondary freshwater fishes and non-native species, above all when reporting similarities in fish populations among drainage systems. There needs to be an explanation why native and non-native species are combined in the cluster analysis. This might generate results that are useful for conservation, but the approach does not provide biogeographic information. The inclusion of historic records and results of recent surveys needs further explanation and discussion.

**Authors:** Dear Reviewer, the non-native species is one of the most critical issue that native species may face, understanding their invasion range within native species range is important to understand and draw management plan. We agree that the inclusion of both historic records and recent survey data requires further explanation, specially this approach may show us the invaded drainage system by non-native species that need more attention. This text is suggested to add into manscript

" The presence and expansion of non-native species is one of the most critical threats facing native freshwater species. Understanding the overlap between the ranges of native and non-native species is crucial for developing effective management and conservation strategies. Therefore, while our primary focus is on geography distribution, we included non-native species in our cluster analysis to highlight their invasion range within native species’ habitats. This approach provides valuable insights into which drainage systems have been most heavily impacted by non-native species, informing targeted management actions.

Furthermore, the combination of historic records and recent survey data allows us to track the temporal dynamics of these invasions. By comparing historical data with recent observations, we can identify drainage systems where non-native species have expanded or established new populations. These areas may require more immediate conservation efforts to protect native species from further displacement or decline."

Here below are more detailed comments. I have also mentioned typos and errors in writing style, because PLOS ONE does not provide copy editing.

**Authors:** We have carefully edited the manuscript and try to remove type errors.

The Abstract is not informative. It should summarize major findings and conclusions.

**Authors:** I added this to Abstract " The eastern drainage systems were dominated by three endemic species, while the western drainage systems had a variety of endemic and non-native species. The study also recorded the presence of non-native fish species in the dams located in Al Baha Region, Abha, and Rabigh, indicating that both eastern and western drainage systems are being invaded by non-native species such as Oreochromis spp. and Carassius spp."

Introduction: An important reference is missing: “Freyhof, J., Els, J. Feulner, G.R., Hamidan, N.A. & Krupp, F. (2020). The Freshwater Fishes of the Arabian Peninsula. Dubai, Motivate Publishing. 272 pp.” This monograph includes the most recently published distribution maps of freshwater fishes native to the Arabian Peninsula.

**Authors:** We have added this reference at the appropriate place in the text.

Another paper that should be added is: Ross, W. (1985). Oasis fishes of Eastern Saudi Arabia. Fauna of Saudi Arabia 7: 303-317.

**Authors:** Added.

References [18] and [19] should also be mentioned in the Introduction.

**Authors:** Done

Methods:

P. 3: It should be mentioned that the two secondary freshwater fish species are Aphaniops dispar and A. stoliczkanus. Further below, Planiliza abu (“Mugil cephalus”) is mentioned as a secondary freshwater fish, which is not correct.

**Authors:** It has been corrected.

The authors state that 16 sites were sampled, with reference to the supporting information provided. However, the supporting information only consists of maps and a table with geographic coordinates without further explanations. The authors should explain what the areas highlighted in red, and the black dots mean and how this information was derived.

**Authors:** The supplementary file has been updated with your suggestion and red area was based on IUNC stander mapping system for freshwater fish specially to estimate (AOO, EOO), I will improve captions of those maps.

Supporting Information: The first map attributed to Mugil cephalus obviously shows the Al-Ahsa record of Planiliza abu. What does the area highlighted in red refer to?

**Authors:** It is " sub-basin" level 6 based on Freshwater Species Mapping Standards for

IUCN Red List Assessments.

For the 16 sites sampled, more detailed information needs to be provided. Were fishes collected, and if so, how? By electric fishing gear, nets and/or visual observations? Has any quantitative method been applied, have specimens been counted by species, size classes, etc.? Have voucher specimens been deposited and, if so, where? Who has done the sampling and how long did the person(s) spend at each sampling site? When were the sites sampled? Dates are important, because wadi fish are usually r-strategists with a very pronounced seasonality in population size and distribution.

**Authors:** Dear Reviewer **, as this work was part of my PhD, please let me know if you agree to keep the current methodology in the paper, as it directly relates to the paper's objectives. Alternatively, if you prefer, I can include a summary of my work general methodology**

**This my general methodology**

**"** The following section provides a description of the basic sampling methods and strategy used for the current study of freshwater fish and fisheries in Saudi Arabia.

## Fieldwork and fish sampling

Between July and September in 2015 (summer), 16 sites in different regions of Saudi Arabia were visited and the fish populations sampled to get an understanding of the diversity of species and freshwater habitats in the country. Five dams, two wadis and one lake were chosen as representative sites for this project. These sites were revisited during winter between 25 December 2015 and 25 January 2016 and again during spring between 28 April 2016 and 19 May 2016. The following fishing methods were used during each fishing trip.

## Sampling strategy

On the first visit, several sampling locations were chosen in each water body and were sampled consistently on each sampling occasion (Figure ‎4.1). Different types of gears most suitable for the conditions encountered were used to catch fish of all sizes where possible (see Section ‎4.3, Fishing gears). Surveys at each location usually took one to two days. A small inflatable boat was used to sample areas that were not easily accessible from the shore, such as in dams.

During each visit, notes were taken of the physical characterization of the water body, observations of human interventions recorded and water quality data collected using standard methods (see Appendix D for details of the form used and Chapters 5, 6, 7 and 8 for details of methodologies used). The following habitat and environmental data were collected: water depth, dam water level, dam length, turbidity, water and air temperature, dissolved oxygen, conductivity, pH, stream characterization and resource users. Fish caught were quickly preserved in 4% formaldehyde and then transported to the laboratory in the United Kingdom for further investigation. The abdominal region of all fish caught was pricked) needle (prior to preservation to ensure the gastrointestinal contents were also preserved. A group of fish (about 30 fish per species, *Carasobarbus apoensis* = 10.9-45.0 cm; *Cyprinion mahalensis* = 9.8-19.0 cm; *Garra buettikeri* = 11.5-15.5 cm; *Oreochromis niloticus* = 10.4-20.0 cm) were also measured (length in mm and weight g) and tagged to track shrinkage in size and weight due to preservation in formalin. Fish based on their location and site preserved in labelled jar and transported to one of the Saudi wildlife labs for preparation of shipping to the United Kingdom. Shipment plastic box were labelled based on location, site, species, total catch weight, number of fishes, data, time were recorded for each sampling trips.

6

4

2

5

3

1

8

7

Figure ‎4.1. Red dots show where fishing gears took place (1 = Thrrad Dam;2 = Al-Janabeen Dam; 3 = Al-Aqiq Dam; 4 = Al-Sadr Dam; 5 = Medhaas Dam; 6 = Al-Asfer Lake; 7 = Wadi Bouah; 8 = Wadi Turbah).

## Fishing gears

More than one fishing method were used at each location to catch fish of different sizes.

Fyke nets: Fyke nets were set in pairs with a 5 m leader net joining them (Figure ‎4.2). The net was green in colour and the mesh size of Fyke nets was 1.5 cm. This net mostly used to catch small and medium-sized fish, although some large fish were also trapped.

Figure ‎4.2. Fyke nets in a green colour similar to this.

Fish traps: Conical fish traps about 1.5 m long with two entrances at the terminal ends were used (Figure ‎4.3). These traps were set in marginal areas and amongst vegetation, and were mostly used to catch small and medium-sized species; the benthic species *Garra* sp. was the species most commonly caught species in the traps.

Figure ‎4.3. Conical fish traps.

Gillnetting: Two sorts of gillnet were used for sampling: single mesh panel gillnets with a 6.35 (cm) mesh size and dual layer trammel nets with mesh sizes of 6.35 and 17.78 (cm). All nets were 30m long and 1.5m deep (hanging ratio E = 0.5). The nets were set overnight in two or three in each site, in the shallow littoral zone or around rocks or tree stumps. In the reservoirs, finding a suitable place to set the nets was challenging because few places less than 5 m deep near the shore were suitable.

Cast netting: Cast netting from the bank was used in some sites, especially wadis, but the gear was ineffective in many sites due to tree branches and turbid water.

Angling: Angling was used on occasions and was found to especially effective during the early morning and to catch various sizes of fishes.

**"**

“Major drainage systems and wadis in Saudi Arabia” should go into the Introduction rather than the Results section.

**Authors:** “Major drainage systems and wadis in Saudi Arabia” **moved to the Introduction.**

P. 3: “Two main natural drainage systems are found in Saudi Arabia”: It should be explained that these drainage basins are in western Saudi Arabia. There are also freshwater bodies in the Central Region and the Eastern Province, but only one species of secondary freshwater fish occurs there. It should be explained that eastern drainage systems end in an endorheic basin.

**Authors:** It has been corrected as suggested.

Caption of Fig. 1: It should be explained what G1 – G4 stands for, which could be done with reference to the text (“groups G1 to G4 explained in the text”). Only M refers to the Al-Madinah Region. Rather than writing that the western wadis drain into the Red Sea, it should be stated that they drain towards the Red Sea (most of them reach the Red Sea only seasonally or not at all, as mentioned further below in the text).

**Authors:** We have corrected it.

P. 3: The Sarawat Mountains are mentioned in the text with reference to Fig. 1. However, the Sarawat Mountains are neither mentioned on the map nor in the caption.

**Authors:** It has been deleted and sentence rephrased.

Throughout the manuscript: The spelling of geographical names must be consistent, e.g. “Wadi Turabah” vs. “Wadi Turbah”.

**Authors:** All the spells have been corrected and now as Wadi Turabah.

P. 4: “Krupp [1982]“ should read “Krupp [1983]”

**Authors:** Done.

P. 4: A wadi is a riverbed, rather than a river valley, usually with a seasonal and/or intermittent water bodies.

**Authors:** Done.

It should be mentioned that relatively high discharge in the southern wadis is due to the monsoonal regime in that part of Saudi Arabia.

**Authors:** It is mentioned in the text.

P. 4: “possibly that may indicate the wadis in this group” should read “possibly indicating that wadis in this group”

**Authors:** Change done.

P 4 and caption of Fig. 1: Al-Madinah or Al-Madinah region should read “Al-Madinah Province” (to distinguish it from the city of Al-Madina Al-Munawwara).

**Authors:** Change done.

P. 4: The sentence “The main wadi channel in the Al-Madinah, Wadi Al-Hemad or Idham” is incomplete, please check.

**Authors:** We have added the word “are” in the sentence to complete it.

P. 5 and Abstract: The term “Biogeographical distribution” does not make sense and needs reconsideration. It should be replaced by “geographic distribution”.

**Authors:** The geographical distribution has been considered throughout the manuscript.

P. 5 “The distribution of indigenous and secondary freshwater fish species” should read “The distribution of indigenous primary and secondary freshwater fish species” (secondary freshwater fish species are also indigenous).

**Authors:** Sentence modified.

P. 5: The heat map is a welcome approach, but more detailed information needs to be provided on how the “density of each fish species group” was calculated, given the fact that many of the published records are very old, published more than 45 years ago, while the samples on which some of these records are based are even older. As stated in the paper, water bodies, ecological conditions, etc., have changed considerably over time and there are many threats that have changed population densities. In many cases, species that have been recorded in the past are likely to be no longer there.

**Authors:** Thank you for your valuable feedback. The density calculations were based on aggregated data from both historic and recent sources, with more weight given to recent records. We acknowledge that older records may no longer reflect current populations due to environmental changes and agree that the figure may be misleading. We propose removing it to avoid potential confusion.

P. 5: The statement “Secondary freshwater fish species include Aphaniops stoliczkanus (Day) and Mugil cephalus.“ needs reconsideration (see comments here above).

**Authors:** Thank you for pointing this out. We acknowledge the need for reconsideration. Upon review, we agree that Aphaniops stoliczkanus and Mugil cephalus do not fit the classification of secondary freshwater species. We will revise the statement accordingly and ensure the species categorization aligns with the correct definitions.

Based on below categories *Mugil cephalus* (Planiliza abu) maybe fit too Complementary

Group.

Primary

Little or no tolerance of saline conditions, e.g. bichirs, pikes,

characins, cyprinids

Secondary

Usually restricted to fresh waters but some can enter or cross from one to another, e.g. garpikes, killifishes, livebearers, cichlids

Diadromous/Anadromous

These spend part of their life cycle in both environments, making most of them very tolerant of changes in salinity, e.g. salmonids

and eels.

Vicarious

Species from a few marine groups that have taken up a mainly freshwater existence, e.g. Lota - the burbot.

Complementary

Migratory members of marine groups that can dominate freshwater habitats in the absence of more specialised freshwater fish, e.g. silversides, mullets, gobies.

Sporadic

These enter freshwater sporadically, can live / breed in either medium, e.g. certain anchovies, mullets, snappers, croakers, sea basses.

P. 5: “biological control” should read “vector control” or “mosquito control”.

**Authors:** Change done.

P 5: “species such Carassius auratus” should read “species such as Carassius auratus”.

**Authors:** Change done.

Throughout the paper: The use of genera spelled out vs. abbreviated is inconsistent and needs attention, e.g. on P. 5 “Garra tibanica, Garra sahilia, C. acinaces, A. arabicus, Acanthobrama hadiyahensis” should read “Garra tibanica, G. sahilia, Cyprinion acinaces, Arabibarbus arabicus, Acanthobrama hadiyahensis” (spell out the genus when first mentioned, followed by an abbreviation, if mentioned again in the same paragraph).

**Authors:** We have thoroughly check manuscript and change has been done where applicable.

Species authors should only be given once upon first mention of a species.

**Authors:** We have checked and corrected where applicable.

P. 6: “Acanthobrama hadiyahensis is endemic to certain wadis” should read “Acanthobrama hadiyahensis is only known from very few water locations”. All of these locations are within the same wadi system. The supplementary map shows 4 locations, 3 of which are mentioned in the text; better list all 4.

**Authors:** Sentence modified.

P.6: “Aphaniops dispar and Aphaniops stoliczkanusis are land-locked marine species” is incorrect; they are both secondary freshwater fishes (which also occur in the sea and other saline water bodies). Better reword: “Aphaniops dispar is a secondary freshwater fish occurring in the western drainages, while A. stoliczkanus is known from the Central Region and the Eastern Province”.

**Authors:** Sentence has been replaced with the suggested one.

P 6: “Ctenopharyngodon Idella” should read “Ctenopharyngodon idella”.

**Authors: Done.**

P. 6: I assume that the record of Garra buettikeri from Wadi Al-Bagarah by Hamidan & Shobrak (2019) is a misidentified G. tibanica or G. sahilia.

**Authors:** This site has been visited and it was G. sahilia**.**

P. 6 and 7: *Aphanius dispar* and *A. stoliczkanus* are not land-locked fish species (see comments here above).

**Authors:** It is corrected with the secondary freshwater fish species.

Throughout: Abbreviations used in the text, such as EP, MR, MRN, need to be explained upon first mention in the text, or a List of Abbreviations should be added.

**Authors:** It has been already given/abbreviated in the legend of the Figure 3.

Fig. 5: “Carassius Carassius” should read “Carassius carassius”.

**Authors:** Map has been updated

Fig. 5: Records of non-native freshwater species are incomplete, Oreochromis mossambicus is missing entirely.

**Authors:** Map has been updated.

P. 7 “tilapia species” should read “tilapiine cichlids”.

**Authors:** Done.

P. 8: “A. hadiyahensis [Critical]” should read “A. hadiyahensis [Critically Endangered]”.

**Authors:** We have revised critical to critically endangered.

P. 8: “are declining” should read “are declining in population size”.

**Authors:** Done.

Discussions: Many statements from the Results section are repeated here without being discussed any further. These statements should be removed. The Discussion requires re-structuring. It may be condensed considerably without losing substance. Some background information included in the Discussion should be moved to the Introduction.

**Authors:**

P. 8: The statement “The distribution of the indigenous freshwater fish fauna appears to be

driven by biogeographical features” does not make sense and needs to be reconsidered. Distribution is always a result of paleogeographic events and ecological conditions.

**Authors:** Correctio Done.

P. 8: “environmental differences between wadis” should read “physical and ecological differences among wadis”.

**Authors:** Correction Done.

P. 8: “Environmental conditions, especially flow regime …” should read “Differences in environmental conditions, especially flow regime …”.

**Authors:** Correction Done.

P. 9: “and have invaded by fish” should read “and have been invaded by fish”.

**Authors:** Corrected.

Throughout the paper: Mugil cephalus should be replaced by Planiliza abu and Aphaniops stoliczkanus is not a marine species.

**Authors:** Mugil cephalus has been replaced by Planiliza abu**.** Marine species has been corrected. Please reply to the 1^st^ sentence.

P. 9: The context of the statement “but these fish are also heavily exploited” is unclear. This probably refers to Ctenopharyngodon idella, but certainly not to Aphaniops stoliczkanus.

**Authors:** “but these fish are also heavily exploited” was deleted from the text.

Information about dams in Saudi Arabia given in the discussion is very important. However, this is background information that needs to go into the Introduction.

**Authors:** We have moved this to the introduction.

The Discussion identifies some needs for further studies in the future, which is very important and more requirements and opportunities for further research should be outlined. The effects of climate change need more consideration.

**Authors:** Thank you for this comment. Future studies will be planned to keep in view of the circumstances found in this study.

P. 11: 2^nd^ sentence of the 2^nd^ paragraph repeats statements made further above in the Discussion and should be deleted.

**Authors:** We have deleted the sentence.

**References**

Throughout the list of References, the use Italics is inconsistent and needs attention.

**Authors:** All the inconsistencies have been resolved.

P. 13: “Al-Ghamdi, H. S., & Abu-Zinadah, O. (1998)” should read “Al-Ghamdi, H. S., & Abu-Zinadah, O.A. (1998).”

**Authors:** Corrected.

P. 13: “Borkenhagen, K. & Krupp, F. (2013). Taxonomic revision of the genus Carasobarbus

karaman, 1971“should read “Borkenhagen, K. & Krupp, F. (2013). Taxonomic revision of the genus Carasobarbus Karaman, 1971” (Karaman is the author of the genus).

**Authors:** Corrected.

The correct and complete citation of [4] is: Coad, B.W., Alkahem, H.F. & Behnke, R.J. 1983. Acanthobrama hadiyahensis, a new species of cyprinid fish from Saudi Arabia. National Museums of Natural Sciences, Publications in Natural Sciences, 2, i–v + 1–6.

**Authors:** Corrected.

Supplementary Table S1. (The locations of water bodies): This needs more explanations. Are these the records from the literature and the sampling sites? If so, the sources (References) and the dates of the visits to the sampling sites should be added. Abbreviations need to be explained.

**Authors:** Table has been updated.
